# Application of metagenomic next-generation sequencing in gastrointestinal infections in children after allogeneic hematopoietic stem cell transplantation

**DOI:** 10.3389/fcimb.2026.1868295

**Published:** 2026-09-03

**Authors:** Zhenghao Xu, Yunzhong Wang, Xin Zhang, Fangzheng Guo, Jie Chen, Haifeng Geng, Yang Li, Yuanyuan Gao

**Affiliations:** 1 Department of Clinical Laboratory, Children’s Hospital of Soochow University, Suzhou, Jiangsu, China; 2 Department of Neonatology, Children’s Hospital of Soochow University, Suzhou, Jiangsu, China

**Keywords:** children, feces, gastrointestinal infections, hematopoietic stem cell transplantation (HSCT), metagenomic next-generation sequencing (mNGS)

## Abstract

**Background:**

Gastrointestinal infections are the leading cause of death for pediatric patients undergoing allogeneic hematopoietic stem cell transplantation (HSCT). Conventional microbiological testing (CMT) often fails to identify the pathogens, resulting in delayed diagnosis and poor treatment outcomes. Metagenomic next-generation sequencing (mNGS) offers a promising method that does not require cultivation, but its application in this specific situation has not been fully studied.

**Methods:**

185 fecal samples were collected from 96 children who underwent HSCT and suffered from diarrhea. All samples were simultaneously subjected to mNGS and CMT testing. The diagnostic performance, pathogen spectrum and prevalence of gastrointestinal infection pathogens were systematically analyzed and compared.

**Results:**

Compared with CMT, mNGS detected significantly more bacteria, viruses and atypical pathogens. Among the pathogens detected by mNGS in the 185 fecal samples, the predominant bacteria were *Pseudomonas* spp. (28 cases), *Clostridioides* spp. (28 cases), *Campylobacter* spp. (25 cases), *Acinetobacter* spp. (16 cases), and *Staphylococcus aureus* (13 cases). *Clostridioides* spp. exhibited a significantly higher detection rate in fecal samples from patients receiving CsA-based combination therapy (p=0.01099) and those with bone marrow from unrelated donors (p=0.03188). *Pseudomonas aeruginosa* (*p* = 0.03038) and *Campylobacter* spp.(*p* = 0.00549) were detected significantly more frequently in patients within the early phase (1–30 days). The detection rate of *Adenovirus* was markedly decreased during the intermediate phase (31–100 days) (*p* = 0.01458). Furthermore, *Polyomavirus* showed a significantly increased detection rate in patients with short-term diarrhea (1–3 days) (*p=*0.03451).

**Conclusion:**

Our findings highlight the substantial superiority of mNGS over CMT in pathogen detection, with a broader coverage encompassing bacteria, viruses, and atypical organisms. It uncovers complex polymicrobial and viral-bacterial co-infections, delineates infection dynamics linked to immune reconstitution. Integrating mNGS into the diagnostic workflow holds great potential for enabling precision antimicrobial therapy and improving outcomes in this high-risk population.

## Introduction

HSCT offers a potentially curative approach for diverse pediatric hematologic malignancies-including acute leukemias, myelodysplastic syndromes (Huynh et al., 2018) and non-malignant conditions such as primary immunodeficiencies, hemoglobinopathies, and inherited metabolic disorders (Sakaguchi et al., 2025).

Despite this progress, the post-transplant period remains characterized by profound, prolonged immunodeficiency that renders patients highly susceptible to infectious complications, a leading cause of non-relapse mortality (Sahin et al., 2016). This immunocompromised state, exacerbated by immunosuppressive therapy for graft-versus-host disease prophylaxis and treatment, places pediatric recipients at elevated risk for opportunistic infections, including viral reactivations, invasive fungal diseases, and encapsulated bacterial pathogens (Norkin et al., 2019). Understanding the kinetics and determinants of immune reconstitution is therefore essential for developing strategies to accelerate immune recovery and reduce infection-related morbidity and mortality in this vulnerable population.

Gastrointestinal infections constitute one of the most challenging and potentially life-threatening complications following pediatric HSCT. Gastrointestinal infections exert devastating consequences on transplant outcomes, patient survival, and quality of life in pediatric HSCT recipients (Gao et al., 2023). Importantly, gastrointestinal infections serve as critical triggers and exacerbating factors for acute GVHD, creating a vicious cycle of mucosal damage, inflammation, and further infection (Chang et al., 2021). The interplay between gastrointestinal infections, GVHD, and transplant-related mortality underscores the critical importance of preventive strategies, early diagnostic approaches, and targeted therapeutic interventions to optimize outcomes in this vulnerable pediatric population.

The accurate and timely diagnosis of gastrointestinal infections in pediatric HSCT recipients remains a formidable clinical challenge. Conventional diagnostic approaches, including fecal culture, acid-fast staining, have long served as the cornerstone for pathogen detection in this vulnerable population. However, the inherent limitations of these traditional methods, particularly regarding detection speed, sensitivity, specificity, and capacity for identifying mixed infections and rare pathogens have become increasingly apparent in the context of modern transplant medicine. The delayed time to diagnosis frequently results in prolonged empiric antimicrobial therapy, contributing to the selection of resistant organisms, increased risk of antimicrobial-related adverse effects, and escalation of healthcare costs. The suboptimal sensitivity leads to underdiagnosis of true infections, potentially resulting in untreated disease progression and increased mortality (Buss et al., 2015). These collective shortcomings underscore the urgent need for more advanced diagnostic platforms capable of overcoming the inherent constraints of conventional approaches, thereby enabling precision medicine-guided management of gastrointestinal infections in this high-risk pediatric population.

The high mortality associated with undiagnosed or misdiagnosed gastrointestinal infections in this vulnerable cohort, combined with the imperative to minimize unnecessary broad-spectrum antimicrobial exposure, necessitates the implementation of more sensitive and comprehensive diagnostic strategies (Miller et al., 2019). The emergence of mNGS has provided a powerful auxiliary tool for the clinical diagnosis and treatment of infectious diseases. Its advantage of not requiring prior prediction of pathogens offers unprecedented potential to overcome the fundamental limitations of conventional detection methods (Goldberg et al., 2015; Gu et al., 2021).

The integration of mNGS into the diagnostic workflow for pediatric HSCT-associated gastrointestinal infections may offer potential advantages for broadening pathogen detection and informing antimicrobial decisions. However, its clinical unlity, performance characteristics, and optimal implementation strategies in this vulnerable pediatric population remain to be systematically defined. Rigorous investigation is warranted to establish whether mNGS can translate improved diagnostic breadth into measurable clinical benefits for this vulnerable pediatric population.

## Methods

### Study site

This study was conducted in Suzhou, a major city in eastern China’s Jiangsu Province with a population of 12.7 million. In 2023, the city had 1.7 million children under the age of 15. The Children’s Hospital of Soochow University (CHSU), situated in central Suzhou, serves as a key pediatric medical center in eastern China and is the sole provincial tertiary children’s hospital in Jiangsu Province. CHSU has 1, 500 beds and annually treats over 70, 000 inpatients and more than 2 million outpatients. This study did not affect patient care and was approved by the Ethics Committee of CHSU (Approval No. 2021CS158). The investigation was conducted in accordance with the guidelines of the Declaration of Helsinki.

### Study design and participants

This study enrolled 96 pediatric patients who developed diarrhea following HSCT for hematologic malignancies at Children’s Hospital of Soochow University between March 2023 and October 2025. A total of 185 feces collected during diarrheal episodes were subjected to mNGS analysis. Inclusion criteria were as follows: (1) increased feces frequency and altered feces consistency following HSCT; (2) absence of severe comorbid systemic diseases; (3) availability of comprehensive medical records.

### Sample collection and processing

Fecal samples from pediatric patients were collected using disposable fecal specimen collection tubes (Sichuan Warrant Biotechnology Co., Ltd.). Portions containing pus, blood, or mucus were selected; if no pus, blood, or mucus was present, samples were collected from multiple sites. The samples were labeled with patient name, hospitalization number, age, gender, and collection time, and then stored at -80°C.

### Conventional microbiological tests

A sterile disposable sampling swab was utilized to immerse the fresh feces of the child into the transport medium before they received any antibiotics, and subsequently inoculated onto Columbia blood agar plate, XLD plate, *Salmonella* enrichment solution (SBG), and alkaline peptone water culture medium (Haibo Biological, China). The inoculated blood plates were incubated in a 5% carbon dioxide 35°C incubator for 24 h, and the growth of bacterial colonies on the XLD plate and enrichment solution was observed in a separate 35°C incubator for 24 h. The *Salmonella* enrichment solution and alkaline peptone water culture medium were incoulated onto XLD plates and No. 4 agar plates, followed by incubation at 35°C for 24 h. If black colonies suggestive of hydrogen sulfide production appear on XLD plates, or if colonies exhibiting black or gray coloration due to potassium tellurite reduction are observed on No. 4 agar plates, identification and confirmation are performed using the MALDI-TOF mass spectrometry.

### Metagenomic next - generation sequencing

Nucleic Acid Extraction: Fecal samples were dissolved in 1 milliliter of physiological saline, then centrifuged at 12, 000g for 5 minutes at 4°C. Next, the sample was added to the Lysing Matrix tube containing Lysing Matrix E, and homogenization was performed using the FastPrep-24™ 5G magnetic bead disruption system and lysis system (Whyte, 2016; Jiang et al., 2022; Li et al., 2022). DNA was extracted from the fecal sample using the QIAamp Circulating Nucleic Acid kit (Qiagen, product number 55114) and the TIANamp magnetic DNA kit (Beijing Tianjin Genomics Company, China). The Agilent 2100 Bioanalyzer system (Agilent, San Jose, USA) was used to assess the quality of the DNA library. After the library was constructed, samples with a DNA content of more than 25 nanograms were sequenced on the Illumina NextSeq 550Dx (Illumina, San Diego, California) platform. Approximately 20 million reads were generated.

### Sequencing and bioinformatics analysis

Pathogen detection through mNGS was accomplished using an internal bioinformatics process, which is the same as that described previously (Zeng et al., 2022; Xu et al., 2023). In brief, first, low-quality reads, adapter contamination, and repetitive and short reads shorter than 36 bases were removed from the sequencing data. Then, human host sequences were identified by mapping human host sequences to the human reference genome (hs37d5) using bowtie2 (Langmead and Salzberg, 2012). Only reads that cannot be mapped to the human genome were retained, and then they were compared with a microbial genome database containing over 12, 000 types of bacteria, over 18, 000 types of fungi, and over 4, 600 types of viruses (downloaded from ftp://ftp.ncbi.nlm.nih.gov/genomes/genbank/) using Kraken2 (v2.0.7) (Wood et al., 2019). The comparison results were matched to complete genomes or chromosome standards at the assembly level.

### Comparison with traditional diagnostic methods

The mNGS pathogen detection process had been previously described (Qian et al., 2020; Liu et al., 2022; Zeng et al., 2022). Positive detections were based on the following criteria: (1) For the detection of *Mycobacterium tuberculosis*, at least one species-specific read was required; (2) For other bacteria (excluding *Mycobacterium tuberculosis*), and viruses, if the species detected by mNGS had at least three non-overlapping read results, then this result was considered positive; (3) If the ratio of microbial reads in each million samples to the negative control (NTC) was <10, then this species was excluded; The pathogens identified by mNGS were classified into three categories: (1) Certain: mNGS results were consistent with CMT results and were consistent with clinical manifestations; (2) Possible: CMT results were either negative or inconsistent with mNGS results, but based on clinical findings, the pathogen detected by mNGS was likely to be the cause of diarrhea; (3) Unlikely: CMT results were either negative or inconsistent with mNGS results, but the pathogen detected by mNGS did not match the clinical manifestations. A true positive was defined as a case in which the clinical presentation could plausibly be caused by the detected pathogen and where at least one diagnostic method yielded a positive result.

### Clinical criteria of intestinal pathogenic bacteria

Among the 185 stool samples, all intestinal pathogens detected by mNGS were evaluated in this study. The causative pathogens responsible for diarrhea were categorized as follows: (1) Well-established enteric pathogens associated with diarrheal disease, including *Clostridioides* spp.*, Campylobacter* spp.*, Salmonella, Mycobacterium, Adenovirus, Cytomegalovirus, Norovirus, Rotavirus.* (2) Species that call into question the appropriability of considering them pathogenic, including *Pseudomonas (*Adlard et al., 1998; Kim et al., 2002; Koh et al., 2018)*, Acinetobacter (*Grotiuz et al., 2006)*, Staphylococcus aureus (*Huang et al., 2017)*, Enterococcus* (Olofsson and Jalakas, 2001)*, Polyomavirus* (Guixia et al., 2012; Pinheiro et al., 2019; Bergallo et al., 2018; Li et al., 2013).

### Statistical analysis

Fisher’s exact test and McNemar’s exact test were used to test the association between categorical variables. Due to the absence of standardized criteria for interpreting mNGS results in this clinical context, and given that our proposed classification framework would likely categorize a substantial proportion of cases as true positives, we refrained from interpreting the sensitivity and specificity of NGS in this study.

## Results

### Demographic and clinical characteristics

A total of 96 pediatric patients with hematologic diseases who underwent bone marrow transplantation were enrolled in this study. Males accounted for 70.83%. Age distribution was mainly concentrated in 6–12 years and 1–3 years. The primary types of hematologic diseases were Acute Myeloid Leukemia (AML), Acute Lymphoblastic Leukemia and Aplastic Anemia (AA). The post-transplantation time windows were relatively evenly distributed across all periods. The main immunosuppressive drugs used after transplantation included cyclosporine A, MMF and Tacrolimus (FK506). Diarrhea occurred predominantly in autumn, summer, and spring. The duration of diarrhea in most pediatric patients was 4–7 days. Twenty-five cases were accompanied by both fever and hematochezia, 18 cases had fever alone, and 17 cases had hematochezia alone. The main sources of donors were parents and unrelated donors. A total of 91.67% of pediatric patients improved, 4 cases were discharged against medical advice, and 4 cases were not cured. The average length of hospital stay was 33.68 days (range: 6–70 days) (**Table 1**).

**Table 1:** Clinical characteristics of patients (n=96).

Characteristic	N (%)
Sex (male)	68 (70.83)
Age (years)	
<1	2 (2.08)
1-3	22 (22.92)
4-5	12 (12.5)
6-12	42 (43.75)
13-18	18 (18.75)
Hematological Disease Classification	
Acute Myeloid Leukemia (AML)	30 (31.25)
Acute Lymphoblastic Leukemia (ALL)	27 (28.13)
Aplastic Anemia (AA)	19 (19.79)
Wiskott-Aldrich Syndrome (WAS)	5 (5.21)
Hemophagocytic Lymphohistiocytosis (HLH)	2 (2.08)
Juvenile Myelomonocytic Leukemia (JMML)	2 (2.08)
Fanconi Anemia (FA)	2 (2.08)
Others/Other diseases	9 (9.38)
Time Window Post-Transplantation	
Early phase (1–30 days)	31 (32.29)
Intermediate phase (31–100 days)	26 (27.08)
Late phase (>100 days)	39 (40.63)
Immunosuppressive Drugs	
Cyclosporine A (CsA)	64 (38.79)
MMF	37 (22.42)
Tacrolimus (FK506)	31 (18.79)
MTX	19 (11.52)
Basiliximab	5 (3.03)
Ruxolitinib	3 (1.82)
Sirolimus	1 (0.61)
Unknown	5 (3.03)
Immunosuppressive protocol	
A single drug	49 (50.59)
Combination of two drugs	23 (27.06)
Combination of three drugs	10 (11.76)
Combination of four drugs	9 (10.59)
Timing of Diarrhea Onset (Seasonal)	
Spring (March–May)	24 (25.00)
Summer (June–August)	27 (28.13)
Autumn/Fall (September–November)	31 (32.29)
Winter (December–February)	14 (14.58)
Duration of Diarrhea	
1–3 days	24 (25.00)
4–7 days	42 (43.75)
8–15 days	20 (20.83)
>15 days	10 (10.42)
Accompanying Symptoms	
Fever	18 (18.75)
Abdominal pain	9 (9.38)
Hematochezia (Blood in feces)	17 (17.71)
Fever + Abdominal pain	14 (14.58)
Fever + Hematochezia	25 (26.04)
Fever + Abdominal pain + Hematochezia	7 (7.29)
None	6 (6.25)
Donor Source	
Unrelated donor	32 (33.33)
Parents	39 (40.63)
Siblings	3 (3.13)
Unrelated and Related	21 (21.88)
Autologous transplantation	1 (1.04)
Outcome	
Improved	88 (91.67)
Self-discharge	4 (4.17)
cured	0 (0)
uncured	4 (4.17)
Mean hospital length of stay (range)	33.68 (6-70)

MTX, Methotrexate; MMF, Mycophenolate Mofetil.

**Figure 1:**
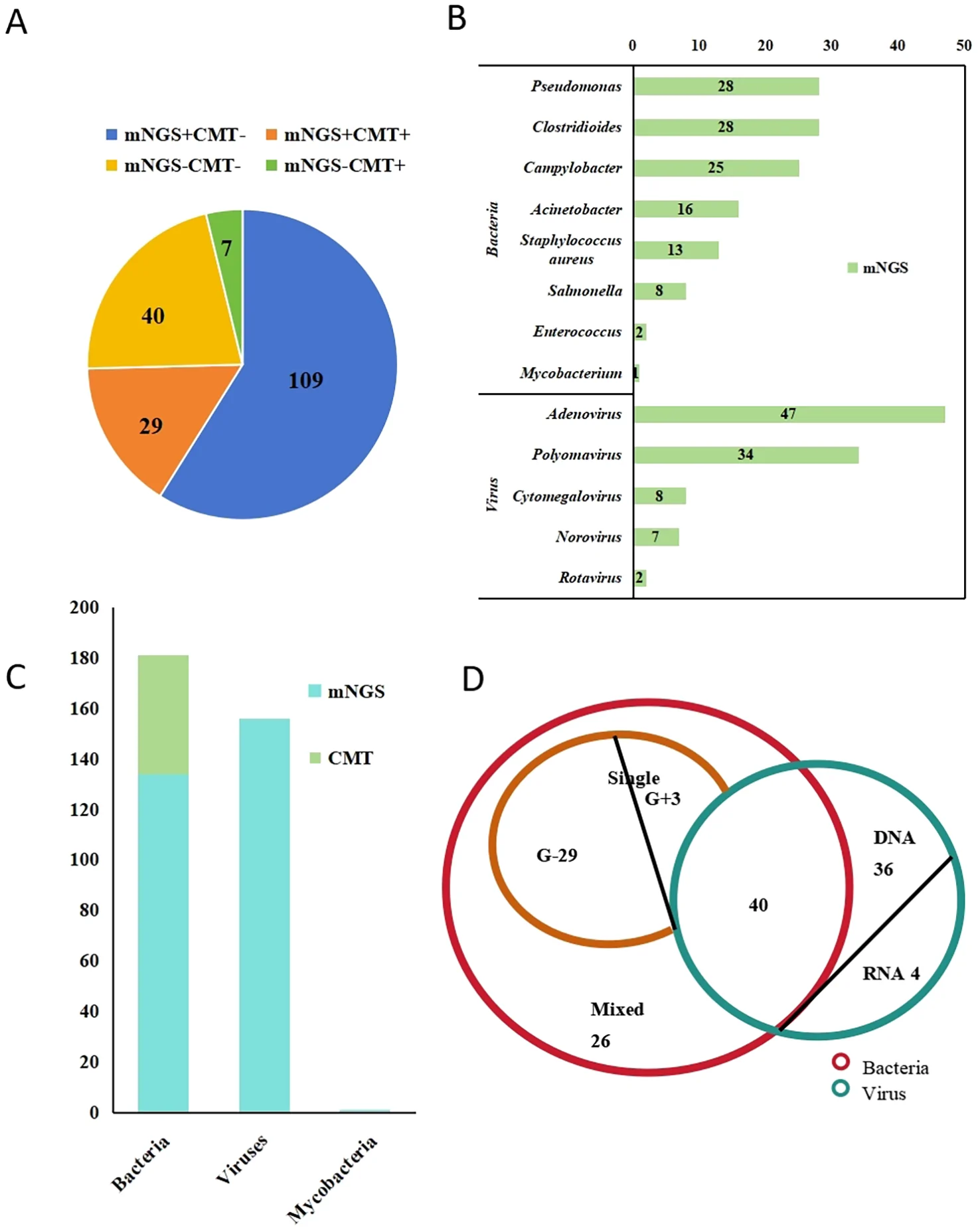
**(A)** A comparison of the positive and negative results of 185 samples detected by mNGS and those detected by CMT. **(B)** Pathogens that tested positive either by mNGS are grouped into two categories: bacteria and viruses. **(C)**The detection rates of mNGS and CMT at the levels of bacteria, viruses and special pathogens. **(D)** The detection of single infections and mixed infections of bacteria and viruses in mNGS positive samples.

A total of 109 cases were positive for mNGS but negative for CMT, 29 cases were positive for both mNGS and CMT, and 40 cases were negative for both (**Figure 1A**). Among the pathogens detected by mNGS in the 185 specimens, the predominant bacteria were *Pseudomonas* spp. (28 cases), *Clostridioides* spp. (28 cases), *Campylobacter* spp. (25 cases), *Acinetobacter* spp. (16 cases), and *Staphylococcus aureus* (13 cases). Regarding viruses, *Adenovirus* (47 cases), *Polyomavirus* (34 cases), *Cytomegalovirus* (8 cases)and *Norovirus* (7 cases) were the most prevalent (**Figure 1B**). Furthermore, mNGS detected significantly more bacterial, viral, and atypical pathogens compared to CMT (**Figure 1C**). Among the 142 pathogen-positive specimens, single bacterial infections were identified in 29 cases, including 26 Gram-negative bacteria and 3 Gram-positive bacteria. Single viral infections were identified in 37 cases, including 34 DNA viruses and 3 RNA viruses. Co-infections included 33 cases of multiple bacteria, 43 cases of bacterial-viral co-infection (**Figure 1D**).

*Clostridioides* spp. exhibited a significantly higher detection rate in fecal samples from patients receiving CsA-based combination therapy (p=0.01099) and those with bone marrow from unrelated donors (p=0.03188). *Pseudomonas aeruginosa* (*p* = 0.03038) and *Campylobacter* spp.(*p* = 0.00549) were detected significantly more frequently in patients within the early phase (1–30 days). The detection rate of *Adenovirus* was markedly decreased during the intermediate phase (31–100 days) (*p* = 0.01458). Furthermore, *Polyomavirus* showed a significantly increased detection rate in patients with short-term diarrhea (1–3 days) (*p=*0.03451) (**Table 2**).

**Table 2:** The relationship between the clinical characteristics of the patients and the pathogenic bacteria detected by mNGS in their fecal samples (n=185).

Clinical characteristics		Specimens, N	*Pseudomonas aeruginosa* N (%)	*Clostridioides* spp. N (%)	*Campylobacter* spp. N (%)	*Adenovirus* N (%)	*Polyomavirus* N (%)
Time Window Post-Transplantation	1–30 days	53	5 (9.43)a	6 (11.32)	13 (24.53)b	14 (26.42)	5 (9.43)
	31–100 days	48	0 (0.00)	4 (8.33)	4 (8.33)	6 (12.50)c	4 (8.33)
	>100 days	84	3 (30.95)	14 (30.95)	8 (30.95)	24 (30.95)	11 (30.95)
Immunosuppressive Regimens	CsA monotherapy	95	3 (3.16)	9 (9.47)	11 (11.58)	18 (18.95)	13 (13.68)
	CsA-based combination therapy	58	4 (6.90)	12 (20.69)d	11 (18.97)	15 (25.86)	4 (6.90)
	Other immunosuppressants	23	1 (4.35)	2 (8.70)	1 (4.35)	8 (34.78)	3 (13.04)
Donor Source	Unrelated	56	4 (7.14)	13 (23.21)e	9 (16.07)	10 (17.86)	4 (7.14)
	Related	86	3 (3.49)	8 (9.30)	11 (12.79)	24 (27.91)	12 (13.95)
	Unrelated and Related	43	1 (2.33)	4 (9.30)	4 (9.30)	10 (23.26)	4 (9.30)
Duration of Diarrhea	1–3 days	40	1 (2.50)	7 (17.50)	6 (15.00)	12 (30.00)	8 (20.00)f
	4–7 days	87	6 (6.90)	12 (13.79)	11 (12.64)	22 (25.29)	7 (8.05)
	8–15 days	36	0 (0.00)	4 (11.11)	4 (11.11)	8 (22.22)	4 (11.11)
	>15 days	22	1 (4.55)	2 (9.09)	4 (18.18)	2 (9.09)	1 (4.55)

^a^*p* = 0.03038, ^b^*p* = 0.00549, ^c^*p* = 0.01458, ^d^*p* = 0.01099, ^e^*p* = 0.03188, ^f^*p* = 0.03451.

## Discussion

The present study provides a comprehensive clinical evaluation of mNGS in diagnosing gastrointestinal infections among pediatric recipients of HSCT. Our findings highlight the substantial superiority of mNGS over CMT in pathogen detection, offering a broader detection range and excelling particularly in identifying mixed infections and fastidious/atypical pathogens.Our study revealed that the post-transplant diarrhea pathogen spectrum was predominantly characterized by conditional pathogens and opportunistic viruses. Bacterial pathogens primarily comprised *Pseudomonas aeruginosa*, *Clostridioides* spp.*, Campylobacter* spp.*, Acinetobacter baumannii*, and *Staphylococcus aureus*, whereas viral pathogens were mainly represented by *Adenovirus*, *Polyomavirus*, *Cytomegalovirus (CMV)*, and *Norovirus*. Notably, mixed infections constituted a substantial proportion, representing a distinctive feature of post-transplant diarrhea. Specific pathogens demonstrated significant associations with immunosuppressive regimens, donor sources, post-transplant temporal phases, and diarrhea duration. The detection rate of *Clostridioides* spp. was significantly elevated in patients receiving cyclosporine and those with unrelated donors. During the early phase (1–30 days), higher detection rates were observed for *Pseudomonas aeruginosa* and *Campylobacter* spp. In the intermediate phase (31–100 days), the detection rate of adenovirus exhibited a significant decline. Furthermore, *Polyomavirus* detection was significantly increased in patients with short-duration diarrhea (1–3 days). These findings underscore the necessity for tailored, risk-stratified preventive and therapeutic strategies in clinical practice.

In pediatric HSCT recipients, gastrointestinal mucosal integrity is frequently compromised due to conditioning regimens and GVHD, characterized by downregulation of tight junction proteins and consequent increased intestinal permeability, which facilitates commensal bacterial translocation (Ara and Hashimoto, 2021). Accordingly, mNGS frequently detects bacteria such as *Pseudomonas aeruginosa, Clostridioides* spp., and *Campylobacter spp*, which may represent colonization, translocation, or active invasion (Balletto and Mikulska, 2015). During the early phase (1–30 days), referred to as the period of delayed immunological reconstitution (Haidrani, 2017) markedly reduced microbial diversity creates an ecological niche that enables *Pseudomonas aeruginosa* to exploit available resources and emerge as a conditional pathogen. *Campylobacter spp*, which localize to the superficial mucosal layer, proliferate under conditions of attenuated gastric acidity and disrupted anaerobic intestinal environment induced by conditioning chemotherapy, thereby establishing themselves as conditional pathogens. Furthermore, the administration of broad-spectrum prophylactic antibiotics alters microbiome architecture, potentially permitting the detection of previously suppressed low-abundance microorganisms.

The incidence of *Adenovirus* infection in children is higher than that in adults (Sedláek et al., 2019). Following HSCT, pediatric patients lacking functional CD4+/CD8+ T lymphocytes experience frequent adenoviral reactivation, rendering *Adenovirus* the most commonly detected viral pathogen in post-transplant diarrhea. The elevated detection rates of *Adenovirus* and *Polyomavirus* during the late phase (>100 days) likely reflect a transition wherein neutrophil counts have recovered while cellular immunity (T-cell function) remains persistently defective; this cellular immune compartment is critical for controlling viral replication and intracellular bacterial pathogens. *Polyomavirus* exhibits low-level intestinal replication and may be associated with transient viral shedding in patients with short-duration diarrhea, resulting in self-limited diarrheal episodes, or alternatively, may represent reactivation of latent virus due to immunosuppression.The pathogenesis of gastrointestinal infections in pediatric HSCT recipients is multifactorial, closely associated with therapy-related gastrointestinal mucosal injury and immune dysfunction (Moreno and Cid, 2019). Conditioning regimens, encompassing high-dose chemotherapy and/or total body irradiation, directly induce cytotoxic damage to gastrointestinal epithelial cells, resulting in mucosal barrier disruption, villous atrophy, and increased gastrointestinal permeability. Cyclosporine, as a calcineurin inhibitor, suppresses the differentiation of T helper 1 (Th1) and T helper 17 (Th17) cells, which constitute pivotal effector subsets for maintaining intestinal epithelial barrier integrity and antibacterial immunity. Conversely, *Clostridioides* spp. exhibit acid resistance and, under conditions of immunocompromise, undergo excessive proliferation with subsequent toxin production. The administration of cyclosporine attenuates host mucosal immune responses against *Clostridioides* spp., impairing bacterial clearance and thereby predisposing to the development of post-transplant intestinal diarrhea. Furthermore, GVHD-associated Paneth cell injury diminishes antimicrobial peptide secretion, thereby compromising mucosal innate immune capacity and facilitating pathogen overgrowth. Our data demonstrate distinct spatiotemporal patterns of gastrointestinal infection across different stages of immune reconstitution (Eriguchi et al., 2012). During the early phase (1–30 days), we observed a predominance of *Campylobacter* spp. and *Pseudomonas aeruginosa*, consistent with the period of neutropenia and mucositis, during which bacteria are prone to translocate across the compromised mucosal barrier.While our study highlights the promise of mNGS, several limitations must be acknowledged. First, the lack of quantitative data in standard mNGS workflows makes it difficult to differentiate between high-load infection and low-load colonization. Although a rigorous multidisciplinary review process was adopted for mNGS report interpretation in this study—integrating microbial detection results with clinical manifestations, laboratory parameters, and direct consultation with the treating physicians—a fundamental diagnostic ambiguity persists in this profoundly immunocompromised pediatric population. In HSCT recipients, conditioning-induced mucosal injury, GVHD-related epithelial damage, and broad-spectrum antibiotic-mediated loss of colonization resistance collectively compromise the intestinal barrier, enabling commensal bacteria and opportunistic pathobionts to translocate into the submucosa or even disseminate hematogenously. Consequently, despite meticulous contextual interpretation at the time of reporting, metagenomic sequencing alone cannot establish whether the detected presence of such organisms represents active infection, passive translocation, or mere colonization, leaving their causal relationship with the patient’s clinical disease fundamentally unresolved. Future studies should explore quantitative mNGS (qmNGS) or digital droplet PCR (ddPCR) validation to establish pathogen load thresholds correlating with clinical disease. Second, the high cost of mNGS currently limits its widespread use; however, as sequencing technologies advance and costs decline, it may become a first-line diagnostic tool. Third, the interpretation of mNGS requires a multidisciplinary team (MDT) comprising transplant physicians, infectious disease specialists, and clinical microbiologists to ensure appropriate therapeutic decisions.

## Conclusion

In conclusion, mNGS is a powerful diagnostic tool that significantly enhances the detection of gastrointestinal pathogens in pediatric HSCT recipients, outperforming conventional methods in both detection rate and pathogen coverage. The integration of mNGS into the diagnostic algorithm holds immense potential to improve clinical outcomes through earlier diagnosis, optimized antimicrobial stewardship, and personalized therapeutic interventions. Further large-scale, prospective studies are needed to standardize reporting criteria and establish definitive correlations between pathogen abundance and clinical severity.

## Data Availability

The original data presented in the study are included in the article/supplementary material, further inquiries can be directed to the corresponding author/s.
